# Impact of handgrip strength on postoperative recovery and prognosis in elderly patients with intertrochanteric fractures

**DOI:** 10.3389/fmed.2025.1724799

**Published:** 2026-01-12

**Authors:** Liling Zhang, Xuan Sun, Haiwu Wan, Bo Huang, Kai Sun

**Affiliations:** 1 Department of Orthopedic Surgery, Jiujiang University Affiliated Hospital, Jiujiang, China; 2 Department of Nursing, Jiujiang University Affiliated Hospital, Jiujiang, China; 3 First Clinical Medical College of Yangtze University, Jingzhou, China; 4 Jiujiang Orthopedic Medical Quality Control Center, Jiujiang, China

**Keywords:** grip strength, intertrochanteric fractures, elderly patients, clinical outcomes, predictor, impact

## Abstract

**Objective:**

To investigate the impact of preoperative grip strength on early postoperative recovery, functional outcomes, and prognosis in elderly patients with intertrochanteric fractures.

**Methods:**

A retrospective cohort study was conducted on 195 elderly patients who underwent closed reduction and intramedullary nailing for intertrochanteric fractures between January 2018 and December 2022. Based on EWGSOP2 cut-off values (men < 27 kg, women < 16 kg), patients were classified into a high grip strength group (*n* = 110) and a low grip strength group (*n* = 85). Data on baseline characteristics, perioperative indicators (postoperative hemoglobin, transfusion volume, time to ambulation, length of hospital stay), postoperative follow-up outcomes (Harris Hip Score, SF-36 quality of life score), as well as postoperative complications and 1-year all-cause mortality were collected and compared between the two groups.

**Results:**

The baseline characteristics were comparable between the two groups (*P* > 0.05). Compared to the high grip strength group, the low grip strength group had significantly lower postoperative hemoglobin levels (7.7 ± 3.4 vs. 9.2 ± 4.1 g/dL, *P* = 0.014), greater postoperative transfusion volume (78.9 ± 44.2 vs. 45.1 ± 26.8 ml, *P* = 0.025), longer time to ambulation (4.2 ± 2.5 vs. 2.5 ± 1.8 days, *P* = 0.034), and extended hospital stay (14.2 ± 3.5 vs. 10.4 ± 4.6 days, *P* = 0.041). At the 6- and 12-month follow-ups, the low grip strength group showed significantly worse Harris Hip Scores and SF-36 scores across all domains (all *P* < 0.05). Furthermore, the incidence of postoperative complications—including pneumonia (9 vs. 4, *P* = 0.033), deep vein thrombosis (6 vs. 4, *P* = 0.010), pressure ulcers (3 vs. 1, *P* = 0.018), urinary tract infections (4 vs. 2, *P* = 0.029), and internal fixation loosening (3 vs. 1, *P* = 0.005)—was significantly higher in the low grip strength group. The 1-year all-cause mortality was also significantly higher in the low grip strength group (4 vs. 2, *P* = 0.040).

**Conclusion:**

Preoperative grip strength is an effective predictor of postoperative prognosis in elderly patients with intertrochanteric fractures. Low grip strength is significantly associated with greater hidden blood loss, a higher incidence of complications, poorer functional recovery, and increased mortality. Incorporating grip strength assessment into the preoperative evaluation and developing targeted rehabilitation strategies may help improve patient outcomes.

## Introduction

Intertrochanteric fractures are among the most common osteoporotic fractures in the elderly, with incidence increasing progressively with age, particularly in individuals aged 65 years and older (1–3). These fractures typically result from low-energy trauma such as falls and are associated with high rates of disability and mortality (4). They significantly impair patients’ quality of life and impose a substantial burden on healthcare resources (5, 6). With the global aging of the population, the incidence of intertrochanteric fractures is expected to rise further (7). Therefore, accurate prognostic assessment and individualized treatment and rehabilitation planning are crucial in clinical practice.

Surgical intervention remains the mainstay of treatment for intertrochanteric fractures. However, postoperative recovery is influenced by multiple factors, including age, sex, bone density, and comorbidities (8–10). The patient’s overall health status, functional capacity, and physiological reserves also play critical roles. Commonly used prognostic tools such as body mass index (BMI), bone mineral density (BMD), and the Charlson Comorbidity Index offer some reference value (11–13). Yet, these indicators do not fully capture the patient’s functional status, particularly in the postoperative period, and often fail to provide effective guidance. Thus, there is a pressing need for a simple, effective, and quantifiable tool for preoperative and postoperative prognostic assessment to aid clinicians in optimizing treatment and rehabilitation strategies.

Grip strength, an important indicator of muscle strength and overall health, has gained increasing attention in geriatric orthopedics (14–16). Declines in grip strength are significantly correlated with frailty, fall risk, hospitalization rates, and mortality (17). Studies have shown that grip strength is related not only to muscle strength, physical function, and balance, but also to metabolic and immune function (18, 19). As a simple, non-invasive, and highly reproducible measure, grip strength is increasingly recognized for its utility in postoperative monitoring and rehabilitation assessment in elderly fracture patients. Growing evidence suggests that grip strength reflects both muscle mass and overall physiological condition (20, 21). Thus, grip strength measurement can provide valuable information for preoperative and postoperative prognostic evaluation. Changes in grip strength are significantly associated with postoperative recovery, functional outcomes, and long-term quality of life (22–24). In elderly populations, early assessment of grip strength is essential for developing individualized rehabilitation plans, monitoring functional recovery, and preventing complications (25, 26).

This study aims to investigate the impact of grip strength on the prognosis of elderly patients with intertrochanteric fractures, specifically examining its relationship with postoperative recovery, functional outcomes, and long-term quality of life. We seek to provide evidence supporting the use of grip strength as a prognostic tool in clinical practice, thereby aiding in the optimization of postoperative management and ultimately improving patient outcomes and quality of life.

## Materials and methods

**FIGURE 1:**
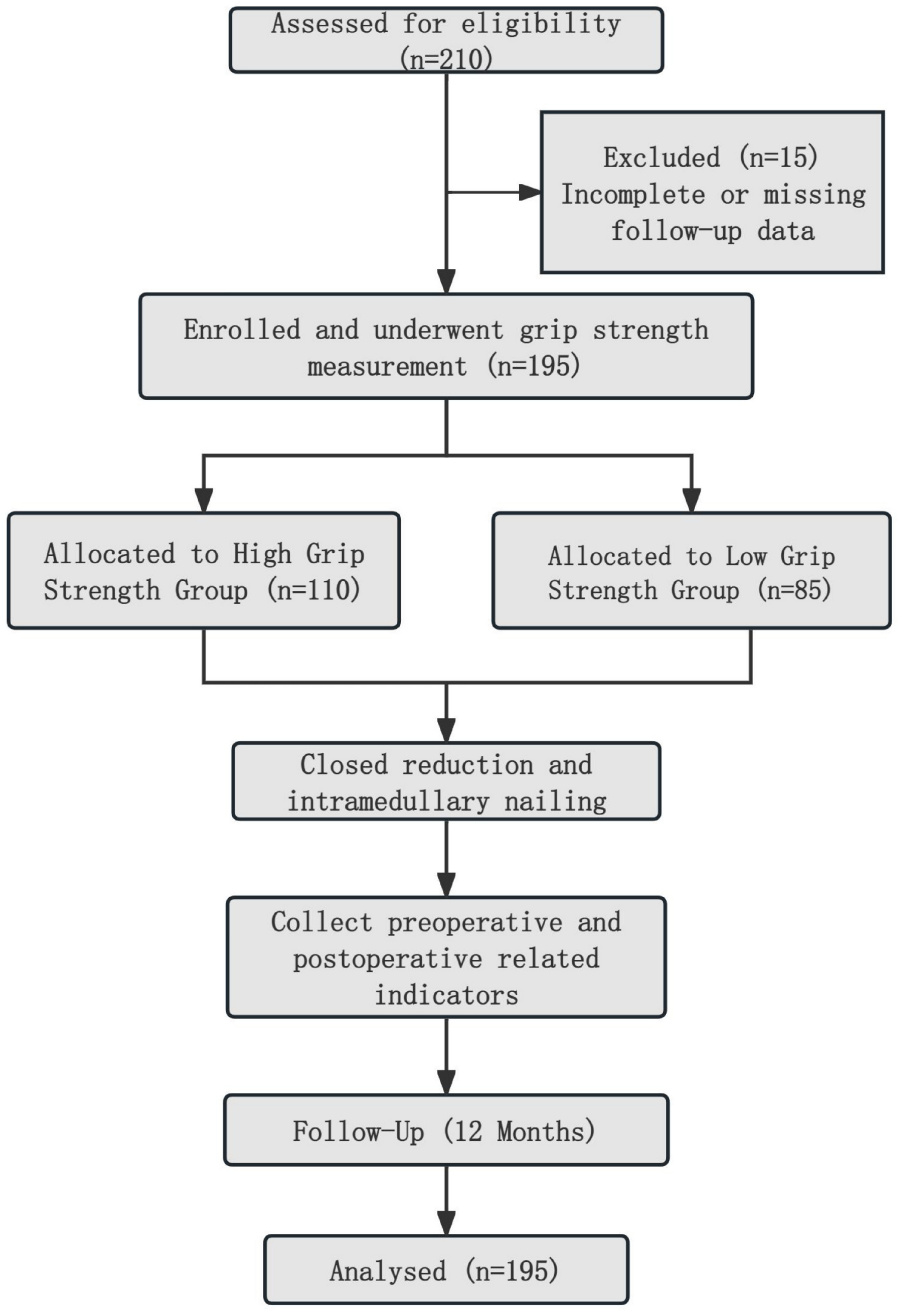
The participant flowchart and research process.

This retrospective study included 210 elderly patients (aged ≥ 65 years) who underwent closed reduction and intramedullary nailing for intertrochanteric fractures between January 2018 and December 2022. The electronic medical records (EMR) of all identified patients were then individually and rigorously screened by two independent researchers against the pre-defined inclusion and exclusion criteria. The detailed screening process and reasons for exclusion are illustrated in the participant flow diagram (Figure 1). Inclusion criteria were: (1) diagnosis of unilateral intertrochanteric femoral fracture; (2) treatment with closed reduction and intramedullary nailing; (3) able to comprehend and cooperate with the grip strength measurement procedure, as determined by a Mini-Mental State Examination (MMSE) score of ≥ 18; and (4) injury resulting from a low-energy fall. Exclusion criteria were: Exclusion criteria were: (1) severe neuromuscular disease or limb dysfunction affecting grip strength measurement; (2) multiple fractures or other severe trauma; (3) high-energy trauma (e.g., motor vehicle accidents or falls from height); (4) severe cognitive impairment, defined as an MMSE score < 18, and 5) incomplete or missing follow-up data.

Grip strength was measured using a calibrated Jamar dynamometer (Carmy EH101). To control for pain variation and its potential impact on grip strength, measurements were taken only after the patient’s acute pain was adequately controlled with analgesic medications. Pain intensity was assessed using a 0–10 numerical rating scale (NRS), and measurements were performed only when the pain score was < 4. The type, dosage, and timing of analgesic administration (e.g., non-steroidal anti-inflammatory drugs or opioids) were systematically recorded to ensure consistency and to account for potential effects on muscle function. Additionally, upper limb edema was assessed, and cases with hand or wrist edema were excluded from the final analysis to avoid confounding factors. Patients were seated with the elbow flexed at 90°and forearm in a neutral position. The dominant hand was tested, beginning with one practice trial followed by three formal measurements, with rest intervals of at least 60 s between trials. The mean value of the three measurements was used for analysis. The assessment followed the standardized protocol recommended by the American Society of Hand Therapists. Based on EWGSOP2 guidelines (27), patients were classified into high or low grip strength groups using cutoffs of 16 kg for females and 27 kg for males. Baseline data collected included age, sex, and BMI. To assess participants’ baseline frailty and pre-injury physical function, the following parameters were evaluated: preoperative mobility level (categorized as unassisted outdoors, assist outdoors/unassisted indoors, indoor assistance, or unable to walk), the Barthel Index (as a measure of activities of daily living), and the Charlson Comorbidity Index (as a measure of comorbidity burden). Fracture type was classified according to the AO/OTA system. Surgical parameters recorded were time to surgery, preoperative hemoglobin level, preoperative albumin level, operative time, intraoperative blood loss, postoperative hemoglobin level, transfusion volume, time to ambulation, and length of stay. Postoperative outcomes included Harris Hip Score, quality of life (SF-36), postoperative complications (e.g., pneumonia, urinary tract infection, deep vein thrombosis, pressure sores), implant-related complications, and 1-year all-cause mortality.

### Surgical procedure

**FIGURE 2:**
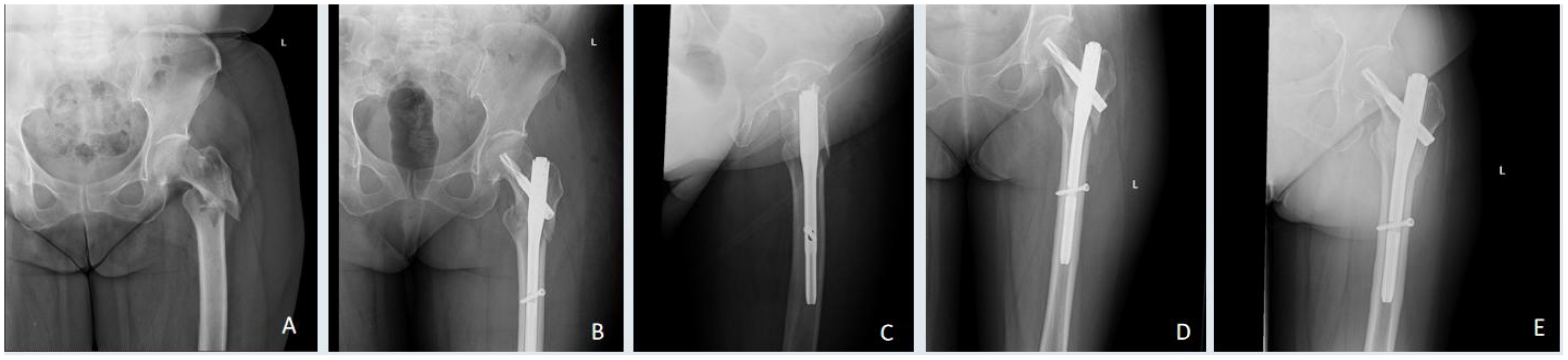
Radiographic follow-up of a patient from the lowgrip strength group. **(A)** Preoperative radiograph showing a left intertrochanteric fracture. **(B,C)** Postoperative radiographs at 2 days. **(D)** Radiograph at 3 months showing callus formation and early union. **(E)** Radiograph at 12 months confirming complete fracture healing.

**FIGURE 3:**
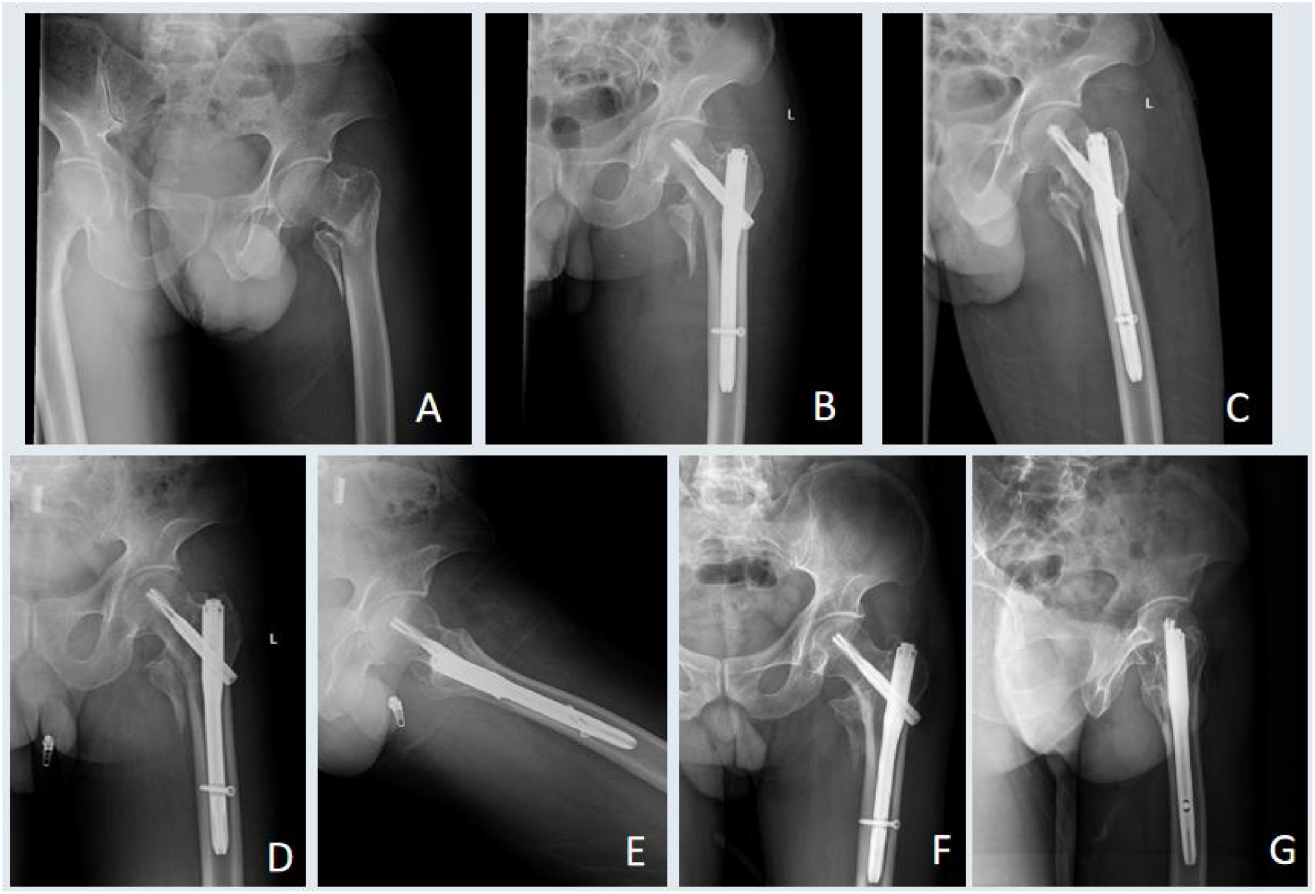
Radiographic follow-up of a patient from the high grip strength group. **(A)** Preoperative radiograph showing a left intertrochanteric fracture. **(B,C)** Postoperative radiographs at 2 days. **(D,E)** Radiographs at 6 months showing advanced fracture healing. **(F,G)** Radiographs at 12 months confirming complete fracture union.

Following successful spinal anesthesia, the patient was placed supine on a traction table. Under C-arm fluoroscopy, closed reduction with traction was performed to achieve satisfactory fracture alignment. After standard skin preparation and draping, a 4 cm longitudinal incision was made over the tip of the greater trochanter. The entry point was exposed, and a guidewire was inserted. After confirming correct placement fluoroscopically, the femoral canal was reamed. An appropriately sized intramedullary nail was inserted, and a guidewire was placed into the femoral neck using a targeting device under fluoroscopic guidance, ensuring central placement in the femoral head and neck with the tip reaching the subchondral bone. A spiral blade was inserted and locked, followed by percutaneous placement of a distal locking screw. Intraoperative fluoroscopy confirmed satisfactory fracture reduction, appropriate implant position and depth, and absence of joint penetration. Cases with favorable healing outcomes during follow-up are presented in Figures 2, 3.

### Perioperative management

All patients received prophylactic antibiotics 30 min before surgery and for 48 h postoperatively. Postoperative pain was managed with short-term non-steroidal anti-inflammatory drugs (NSAIDs). Low molecular weight heparin was administered for deep vein thrombosis prophylaxis based on Autar scores. Patients were encouraged to mobilize early with walking aids, and both groups participated in early rehabilitation under the guidance of a physical therapist.

### Postoperative complication diagnosis and verification

**FIGURE 4:**
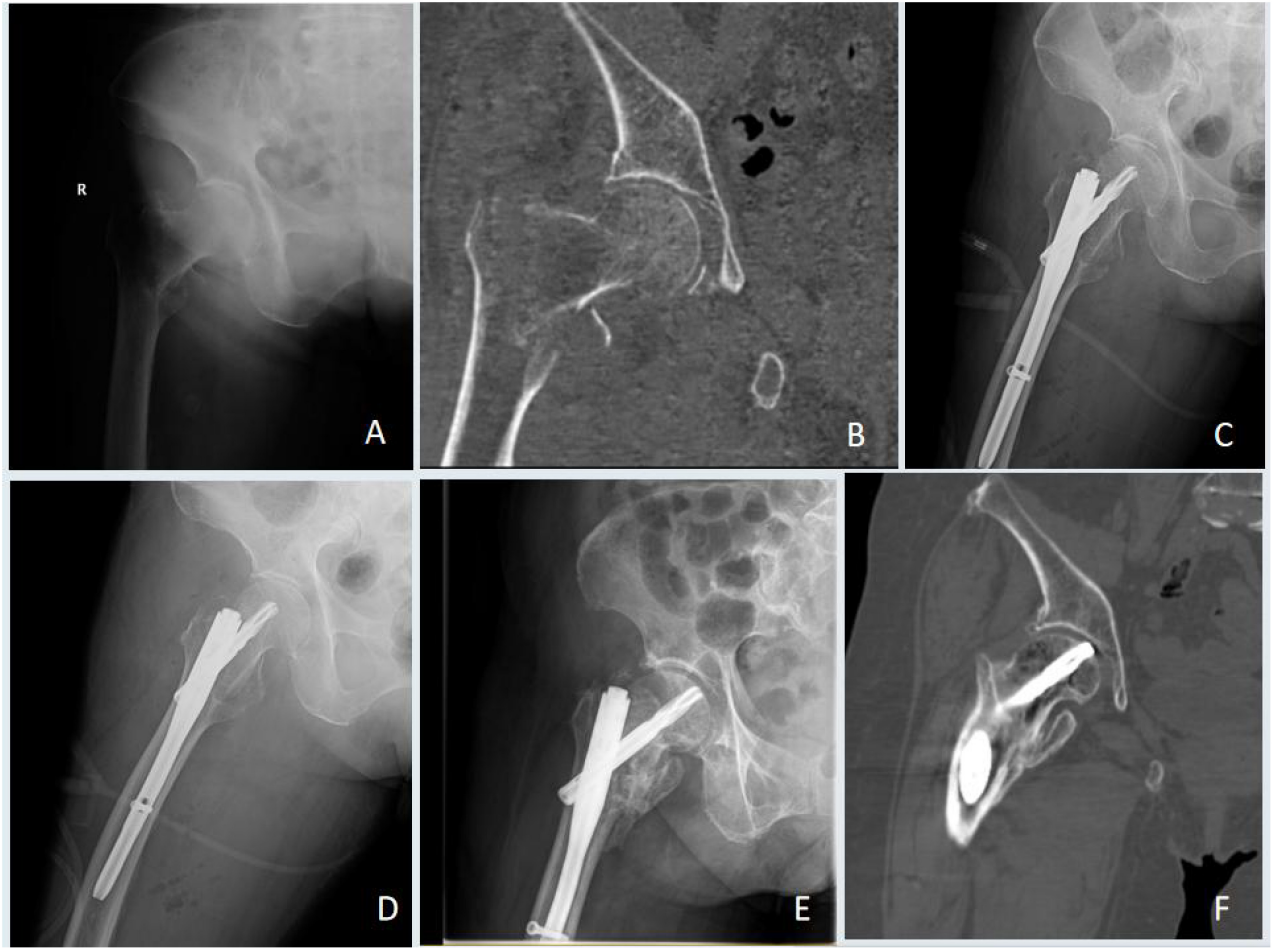
Implant-related complication (screw cut-out) in a patient from the low grip strength group. **(A,B)** Preoperative radiographs showing a right intertrochanteric fracture. **(C,D)** Postoperative radiographs at 2 days. **(E,F)** Follow-up radiographs at 12 months demonstrating fracture healing with concomitant femoral neck shortening and partial screw cut-out (arrow).

**FIGURE 5:**
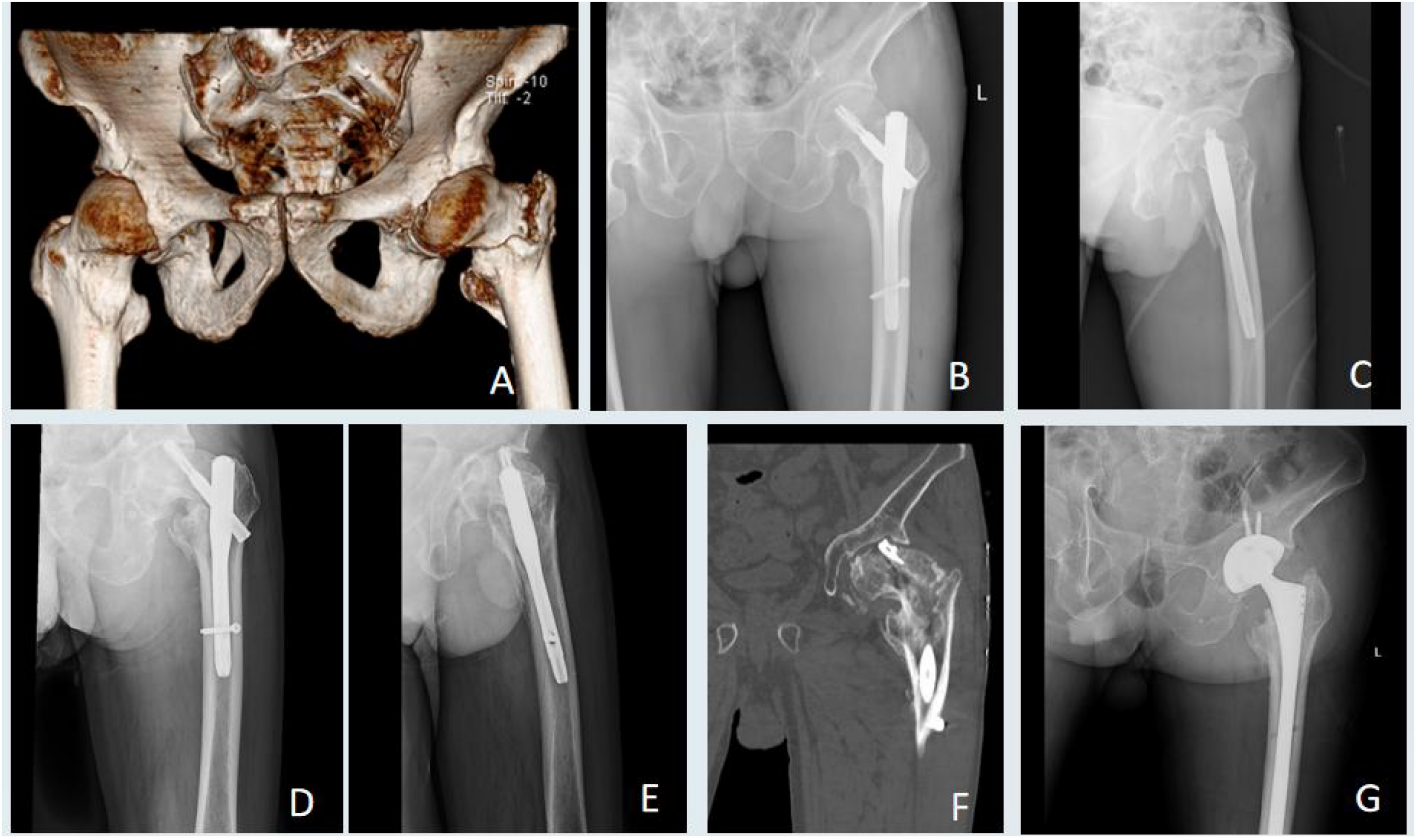
Management of a major implant failure in a patient from the low grip strength group. **(A)** Preoperative radiograph showing a left intertrochanteric fracture. **(B,C)** Postoperative radiographs at 2 days. **(D–F)** Radiographs at 6 months revealing implant cut-out and failure. **(G)** Radiograph after implant removal and subsequent total hiparthroplasty.

Pneumonia: Diagnosed per CDC criteria, requiring new pulmonary infiltrates on imaging plus at least two supporting clinical symptoms. Deep Vein Thrombosis (DVT): Confirmed by compression duplex ultrasonography performed by a certified radiologist. Pressure Injury: Assessed and staged daily by nursing staff using the Braden Scale and NPUAP staging system. Urinary Tract Infection (UTI): Diagnosed based on a positive urine culture, pyuria, and relevant clinical symptoms. Implant Loosening: Defined as a progressive radiolucent line > 2 mm around the implant or a clear change in position on serial radiographs, assessed by two independent orthopedic surgeons. (Typical cases of implant loosening are shown in Figures 4, 5.

### Functional outcome assessment

Functional outcomes, including the Harris Hip Score and the SF-36 quality of life survey, were assessed at 6 and 12 months postoperatively. All assessments were conducted by a dedicated research physical therapist who was blinded to the patients’ grip strength group allocation. A standardized protocol was followed for the administration of the HHS, which included a structured interview and a physical examination of hip function. The SF-36 was self-administered by patients in a quiet environment with assistance available if required for clarification.

### Sample size and statistical analysis

*A priori* power analysis (G*Power; repeated-measures ANOVA, α = 0.05, power = 0.9) determined that 45 patients per group were required to detect differences in functional outcomes. The final sample size (High: *n* = 110; Low: *n* = 85) exceeded this requirement, ensuring adequate statistical power. Data were analyzed using SPSS version 23.0 (IBM Corp., Armonk, NY, United States). For the primary analysis of functional outcomes (Harris Hip Score and SF-36), a complete-case analysis was initially employed. A worst-case scenario sensitivity analysis was performed for patients who died or required revision surgery, assigning the worst possible scores (0 for HHS and SF-36) from the time of the event onward. A complete-case analysis was employed as the data missing rate for all variables was very low (< 2%) and judged to be missing completely at random (MCAR). Continuous variables were compared using Independent Samples *t*-test or Mann-Whitney U test, and categorical variables using Chi-square or Fisher’s exact test. To control for potential confounders, Harris Hip Score were further analyzed using multivariable logistic regression. Models were adjusted for age, sex, Charlson Comorbidity Index, and preoperative albumin level. A *p* < 0.05 was considered statistically significant.

## Results

A total of 195 patients were included in the final analysis: 110 in the high grip strength group and 85 in the low grip strength group. Baseline characteristics did not differ significantly between groups (*P* > 0.05) (Table 1). Preoperative hemoglobin, Preoperative albumin level, operative time, and intraoperative blood loss were also comparable (*P* > 0.05). However, significant differences were observed in postoperative hemoglobin levels, transfusion volume, time to ambulation, and length of stay (*P* < 0.05) (Table 2). At 6 and 12 months postoperatively, both Harris Hip Scores and SF-36 scores showed statistically significant differences between groups in the complete-case analysis (Table 3). To address potential bias from missing data, a worst-case scenario sensitivity analysis was conducted. This analysis included imputing the worst possible outcomes for patients who died or developed major complications before assessment. The results of this sensitivity analysis corroborated the primary findings, with all functional outcomes remaining significantly better in the high grip strength group at 12 months (all *p* < 0.05). Several postoperative complications also differed significantly (*P* < 0.05), as did the 1-year all-cause mortality rate (*P* < 0.05) (Table 4). Multivariate regression identified that higher grip strength, a lower Charlson Comorbidity Index (CCI) score, and a higher pre-fracture Barthel Index were independent predictors of a higher Harris Hip Score at 12 months postoperatively (*P* < 0.05) (Table 5).

**TABLE 1:** Comparison of baseline characteristics of patients.

	High grip strength (*n* = 110)	Low grip strength (*n* = 85)	*P*-value
Age (years)	76.1 ± 7.9	78.4 ± 10.2	0.670
Gender (male/female)	34/76	23/62	0.125
BMI (kg/m^2^)	23.0 ± 3.8	21.9 ± 5.7	0.516
Barthel index (before injury)	77.9 ± 25.3	75.5 ± 31.2	0.074
**CCI groups**			
No comorbidity/mild	38	30	0.258
Moderate	66	51	0.680
Severe	6	4	0.097
**AO/OTA classification**			
31A1	74	55	0.895
31A2	28	23	0.319
31A3	8	7	0.452
**Mobility level prior to fracture**			
Unassisted outdoors	68	52	0.090
Assist outdoors, unassisted indoors	31	24	0.685
Indoor assistance	10	8	0.217
Unable to walk	1	1	0.087

**TABLE 2:** Comparison of perioperative indexes.

	High grip strength (*n* = 110)	Low grip strength (*n* = 85)	*P*-value
**Surgery waiting time**			
48–72 h	53	40	0.771
<24 h	25	21	0.215
>72 h	32	24	0.931
**Preoperative indicators**			
Preoperative hemoglobin (g/dL)	11.5 ± 3.8	11.1 ± 4.5	0.482
Preoperative albumin (g/dL)	3.8 ± 0.82	3.6 ± 0.69	0.102
Operation time (min)	34.5 ± 15.2	32.1 ± 12.7	0.142
Intraoperative blood loss (mL)	25.9 ± 21.2	23.2 ± 22.8	0.228
**Postoperative indicators**			
Postoperative hemoglobin (g/dL)	9.2 ± 4.1	7.7 ± 3.4	0.014
Postoperative blood transfusion volume (mL)	45.1 ± 26.8	78.9 ± 44.2	0.025
Time to ambulation (days).	2.5 ± 1.8	4.2 ± 2.5	0.034
Hospital stay (days).	10.4 ± 4.6	14.2 ± 3.5	0.041

**TABLE 3:** Comparison of postoperative follow-up.

	High grip strength (*n* = 110)	Low grip strength (*n* = 85)	*P*-value
**Harris Hip Score**			
6 months postoperatively	72.7 ± 8.6	65.2 ± 10.8	0.019
12 months postoperatively	80.5 ± 5.9	72.8 ± 8.7	0.021
**SF-36 quality of life score**			
**SF-36 physical health**			
6 months postoperatively	30.8 ± 3.6	28.5 ± 4.4	0.037
12 months postoperatively	35.4 ± 2.7	31.8 ± 4.8	0.016
**SF-36 mental health**			
6 months postoperatively	32.5 ± 4.8	30.8 ± 5.3	0.023
12 months postoperatively	35.9 ± 4.2	33.7 ± 4.1	0.008

**TABLE 4:** Comparison of postoperative complications and all-cause mortality.

	High grip strength (*n* = 110)	Low grip strength (*n* = 85)	*P*-value
**Postoperative complications**			
Pneumonia	4	9	0.033
Deep vein thrombosis	4	6	0.040
Pressure ulcers	1	3	0.038
Urinary tract infections	2	4	0.029
Implant loosening	1	3	0.015
All-cause rate within 1 year after surgery	2	4	0.042

**TABLE 5:** Univariate and multivariate analysis for variables significantly associated with 12 months postoperatively Harris Hip Score.

Predictors	Univariate analysis		Multivariate analysis	
	β (95% CI)	*p*-value	β (95% CI)	*p*-value
Age	−0.13(−4.25 to 2.44)	0.106		
BMI	−0.12(−12.26 to 1.02)	0.073		
CCI	−1.50 (−2.20 to 0.89)	<0.001	−1.20 (−1.92 to 0.54)	0.018
Barthel index (before injury)	0.32 (0.15 to 0.49)	< 0.001	0.25 (0.08, 0.42)	0.030
Fracture classification	0.23(0.14 to 4.03)	0.029	0.36(−0.22 to 3.48)	0.503
HGS	0.25 (0.11 to 0.49)	<0.001	0.22 (0.07 to 0.37)	< 0.001
Surgery waiting time	−0.23 (−0.70 to 0.24)	0.073		
Preoperative hemoglobin	0.63(−3.93 to 7.81)	0.598		
Preoperative albumin level	−0.05(−1.78 to 0.77)	0.326		
Operation time	0.23(−1.83 to 3.49)	0.073		
Time to ambulation	0.06(−4.91 to 12.82)	0.314		
Hospital stay	−0.15(−0.82 to 0.09)	0.467		

## Discussion

Declines in muscle strength significantly impair the body’s ability to recover from injury, including surgical trauma. Sarcopenia has increasingly been recognized as a predictor of surgical outcomes and is associated with postoperative complications and mortality in geriatric orthopedic patients (28–30). Traditional methods for assessing sarcopenia are often complex and impractical for routine clinical use. This study employs grip strength as a simple, convenient, and clinically feasible surrogate for sarcopenia evaluation.

Covert blood loss is a common yet frequently overlooked issue after intertrochanteric fracture surgery, with important implications for recovery. We found that patients with low grip strength had greater covert blood loss and required more postoperative transfusions. While previous studies have identified factors such as BMI, operative time, fracture type, and medication use as influencers of covert blood loss (31, 32). However, our study identified a significant association between grip strength, and covert blood loss as well as postoperative transfusion requirements in elderly patients with intertrochanteric fractures. A study indicated that elderly patients with significantly lower preoperative grip strength in intertrochanteric fracture surgery had an average increase of 15–20% in covert blood loss, along with a marked increase in transfusion requirements. Further analysis revealed that these patients experienced a more significant decline in postoperative hemoglobin levels, with a notably longer recovery time to preoperative levels (33). This suggests that lower grip strength is not only directly related to postoperative blood loss but may also affect the speed of recovery in the blood system. Similar findings were reported in a study on hip arthroplasty, where patients with low grip strength had a significantly higher transfusion rate compared to those with normal grip strength (12.5% vs. 24.4%) (34). Another study on sarcopenia and femoral proximal fractures found that lower total body muscle density was associated with higher transfusion requirements, indicating a link between sarcopenia and the need for transfusions (35). The underlying mechanisms may involve the systemic physiological decline associated with low grip strength. A weakened metabolic and inflammatory profile could exacerbate the pathophysiological processes of covert blood loss, such as capillary leakage and impaired coagulation function, thereby increasing postoperative transfusion demands (36, 37). The reason for the increased transfusion demand in patients with low grip strength may be due to a weakened overall metabolic function and altered inflammatory status, which increase the risk of capillary leakage and blood loss.

One of the findings of this study is the association between postoperative DVT incidence and grip strength. A multicenter retrospective study indicated that patients with lower grip strength have a 40% increased risk of developing DVT, particularly in the early postoperative phase (38). An association between lower grip strength and an increased incidence of postoperative deep vein thrombosis (DVT) was another key finding. This can be interpreted as a clinical manifestation of underlying sarcopenia. The systemic muscle weakness it entails likely impairs the calf muscle pump, reducing venous return and creating stasis in the lower limbs (39). Another major factor is the inflammatory response and endothelial dysfunction. Low grip strength is a marker of sarcopenia and is closely associated with an enhanced systemic inflammatory state (40). Elevated levels of inflammatory markers have been shown to increase the risk of thrombus formation. Inflammatory factors can also induce endothelial dysfunction, disrupting normal blood flow and promoting thrombosis (41).

Postoperative functional recovery is a crucial goal for elderly patients with intertrochanteric fractures. Our data demonstrate a significant correlation between preoperative grip strength and functional outcomes, as measured by the Harris Hip Score and SF-36. This association can be explained by several factors. Patients with greater grip strength, indicative of overall robustness, likely possess better lower limb strength and balance, facilitating safer and earlier ambulation. Furthermore, they may exhibit greater resilience and adherence to postoperative rehabilitation protocols, thereby optimizing their functional recovery (42, 43). This may be related to better muscle coordination and psychological resilience in patients with higher grip strength. Conversely, patients with lower grip strength are more prone to functional impairments after surgery, especially gait abnormalities and balance deficits, which restrict their daily activities. Grip strength may also influence patients’ adherence to rehabilitation training. Patients with higher grip strength are generally more capable of completing postoperative physical therapy and exercise regimens, whereas those with lower grip strength may interrupt rehabilitation due to fatigue, pain, or lack of motivation (44). When interpreting the statistical significance of individual complications listed in Table 4, caution is warranted due to the low incidence rates of some events. For certain complications, such as pressure ulcers and implant loosening, the absolute differences between groups, although statistically significant (*P* < 0.05), are based on a small number of events (e.g., 1 vs. 3). In such cases, the *p*-value, while indicative of a potential association, should not be overinterpreted regarding its clinical impact without support from larger studies. Therefore, the findings for these specific low-incidence complications should be considered exploratory and hypothesis-generating. The principal conclusion from our analysis of postoperative complications is not reliant on any single outcome but rather on the consistent and robust trend observed across multiple domains, indicating a higher overall burden of complications in the low grip strength group. Studies have indicated that the interruption rate of postoperative rehabilitation is significantly higher in patients with low grip strength, which may further worsen their postoperative functional recovery outcomes (45). The lower incidence of postoperative complications in our cohort compared to some literature may reflect our stringent diagnostic criteria requiring objective confirmation, implementation of a standardized perioperative care protocol, and the exclusion of patients with severe cognitive impairment—potentially selecting a healthier patient population.

Grip strength, as a comprehensive indicator of overall health, is closely related to patients’ prognosis. This study demonstrates that patients with lower preoperative grip strength have a significantly higher 1-year all-cause mortality rate and a higher incidence of implant failure. Similar studies have reported that the 1-year mortality rate for patients with low grip strength is 20%, compared to only 10% for those with normal grip strength (46). This result is thought to be closely associated with malnutrition and an inflammatory state in patients with low grip strength. Furthermore, this study found that patients with lower grip strength are more prone to implant failure. Some studies suggest that grip strength is closely related to overall muscle strength, particularly the strength of the upper limbs and core muscles (20, 47). Muscle weakness can impair balance and coordination, increasing stress on the implant, and thereby increasing the probability of implant failure. Other studies indicate that a decline in grip strength is a common manifestation of sarcopenia, which is associated with delayed fracture healing and an increased risk of complications (48–50). Therefore, patients with lower grip strength may face a higher risk of implant failure.

Although the aforementioned studies have provided several new insights, this study has some limitations. First, the sample size in this study is relatively small, which may affect the generalizability of the results. Future research could involve larger sample sizes, multicenter, and prospective studies to further validate the causal relationship between grip strength and the outcomes of elderly patients with intertrochanteric fractures. A further limitation is potential bias from informative censoring. Future studies should use advanced methods like mixed-effects models to handle missing data. While multivariable models were used to control for potential confounders, residual confounding from unmeasured factors (e.g., specific medication details) inherent to observational studies cannot be completely excluded.

## Conclusion

There were significant differences in postoperative hip function (Harris score) and quality of life (SF-36 score) between the high and low grip strength groups. The high grip strength group had a lower incidence of postoperative complications and a significantly lower all-cause mortality rate compared to the low grip strength group. A specially designed rehabilitation training program, implemented both preoperatively and postoperatively, may effectively improve patients’ grip strength, thereby enhancing the overall postoperative recovery of patients with intertrochanteric fractures.

## Data Availability

The raw data supporting the conclusions of this article will be made available by the authors, without undue reservation.
